# Genome-Wide Identification of the Whirly Gene Family and Its Potential Function in Low Phosphate Stress in Soybean (*Glycine max*)

**DOI:** 10.3390/genes15070833

**Published:** 2024-06-25

**Authors:** Zhimin Li, Xuhao Zhai, Lina Zhang, Yifei Yang, Hongqing Zhu, Haiyan Lü, Erhui Xiong, Shanshan Chu, Xingguo Zhang, Dan Zhang, Dandan Hu

**Affiliations:** 1 Collaborative Innovation Center of Henan Grain Crops, College of Agronomy, Henan Agricultural University, Zhengzhou 450046, China; lzmzdb@126.com (Z.L.); zhaixuh@163.com (X.Z.); 18438707561@163.com (L.Z.); yangyifei_11@stu.henau.cn (Y.Y.); zhuhongqing2021@163.com (H.Z.); xiongerhui@henau.edu.cn (E.X.); chushanshan@henau.edu.cn (S.C.); xingguozhang@henau.edu.cn (X.Z.); zhangd@henau.edu.cn (D.Z.); 2 College of Information and Management Science, Henan Agricultural University, Zhengzhou 450046, China; lvhaiyan0319@163.com

**Keywords:** whirly, expansion, expression patterns, haplotype, phosphate deficiency

## Abstract

The *Whirly* (*WHY*) gene family, functioning as transcription factors, plays an essential role in the regulation of plant metabolic responses, which has been demonstrated across multiple species. However, the *WHY* gene family and its functions in soybean remains unclear. In this paper, we conducted genome-wide screening and identification to characterize the *WHY* gene family. Seven *WHY* members were identified and randomly distributed across six chromosomes. The phylogenetic evolutionary tree of *WHY* genes in soybean and other species was divided into five clades. An in-depth analysis revealed that segmental duplications significantly contributed to the expansion of *GmWHYs*, and the *GmWHY* gene members may have experienced evolutionary pressure for purifying selection in soybeans. The analysis of promoter Cis-elements in *GmWHYs* suggested their potential significance in addressing diverse stress conditions. The expression patterns of *GmWHYs* exhibited tissue-specific variations throughout the different stages of soybean development. Additionally, six *GmWHY* genes exhibited different responses to low phosphate stress. These findings will provide a theoretical basis and valuable reference for the future exploration of *WHY* gene function.

## 1. Introduction

Whirlies (WHYs) are a group of plant-specific proteins that can be found in various organisms and are mainly present in organelles and nuclei [1]. WHY proteins are a type of DNA/RNA-binding protein that can be found in many areas of the plant cell and play crucial roles in plant nuclei and organelles [2]. They exhibit a remarkably conserved WHY DNA-binding domain and possess a shared KGKAAL motif within this domain that enhances their affinity for single-stranded DNA [3]. WHY-like proteins without the KGKAAL motif can also be observed in green algae species, like *Ostreococcus tauri* [4]. While the presence of a single WHY-like protein is observed in algae, higher plant species exhibit a minimum of two WHY proteins. Both possess a peptide that guides them to either mitochondria, plastids, or both organelles [5].

In plants, WHY1 was initially recognized as the subunit of a 24 kDa protein that binds to the promoter region of the PR-10a gene in potato [6]. Crystal analysis results showed that WHY1 consists of a spiral structure formed by the interaction of four p24 molecules, which are connected through a motif characterized by a helix–loop–helix pattern [7]. A comparative analysis of WHY proteins from potato and *Arabidopsis thaliana* demonstrated the existence of a conserved arrangement of distinct amino acids, which serves as a foundation for the non-sequence-specific binding of single-stranded DNA [8]. There are two types of WHY proteins in most higher plants, but *A. thaliana* and other *Brassicaceae* family members exhibit three distinct WHIRLIES. Among these, WHY1 is specifically directed towards chloroplasts, while WHY2 targets mitochondria and WHY3 demonstrates a dual targeting ability for both organelles [3,5].

The role of *WHY*s is essential in the control of gene expression as they act as transcription factors [9]. They can bind to specific DNA sequences and exert regulatory control over the activation or suppression of target genes. This regulatory function enables plants to modulate their patterns of gene expression in response to various environmental stimuli, including light, temperature fluctuations, and stress signals [3,10]. In recent decades, *WHY*s have been studied and their functions have been found to be diverse and essential for the growth, development, and survival [9,11,12]. Leaf variegation was found in the progeny of a *why1*/*why3* double knock out mutant, indicating interference in chloroplast development in *A. thaliana* [13]. In maize, *ZmWHY1* transposon mutants may have ivory or albino leaves due to the lack of plastid ribosomes, ultimately leading to their demise once they reach the stage of developing three or four leaves [12]. The hybrid between *Zmwhy1* and *Zmemb16* showed impaired embryonic development and albino seedlings [14]. A barley variety with reduced levels of WHY1 exhibited a slower rate of ribosome formation and chloroplast maturation [15].

*WHY*s are not only involved in chloroplast development, but also in the regulation of organ senescence. The *why1* mutant exhibited premature aging characteristics in *A. thaliana*. AtWHY1, an upstream modulator of the senescence-inducing transcription factor WRKY53, has the ability to bind to the *WRKY53* promoter, inhibit the expression of *WRKY53*, and postpone leaf senescence [16]. Having received growing interest in recent years, the involvement of WHY proteins that are specific to plants has been increasingly recognized in both developmental processes and the enhancement of stress tolerance. However, there are few reports about the systematic studies and function of WHY proteins in soybeans.

Soybean (*Glycine max*), as a crucial economic and oil crop, holds immense significance in various aspects of human life, which is not only be used for direct consumption but also serves as a fundamental protein source for both humans and animals [17,18]. However, the growth and development process of soybeans can be greatly affected by various abiotic stresses, leading to a significant impact on their overall yield. These abiotic stresses include salinity and phosphate (Pi) deficiency, etc. [19,20]. Therefore, it is essential to systematically identify and examine the *Whirly* gene family to understand and explore its biological functions in soybean.

In this research, the soybean *Whirly* gene family (*GmWHYs*) was identified, and a bioinformatics analysis as well as expression analysis were conducted to gain systematic insights into their composition, classification, and expression characteristics. Subsequently, we investigated the alterations of *GmWHY* member expression levels under low Pi stress. Our findings could provide a foundation for further understanding the biological functions of *GmWHY*s in soybean’s reaction to low Pi stress.

## 2. Materials and Methods

### 2.1. Identification of Putative GmWHY Genes in Soybean

The *WHY* gene sequences in *A. thaliana* were obtained from the TAIR database (https://www.arabidopsis.org/ (accessed on 20 December 2023)) and utilized for conducting a basic local alignment search (BLAST) within the soybean genome database (https://phytozome-next.jgi.doe.gov/info/Gmax_Wm82_a2_v1 (accessed on 20 December 2023)). The HMM profiles of the Whirly family (PF08536) was acquired from the Pfam database, accessible at http://pfam.xfam.org/ (accessed on 20 December 2023). The hmmSearch tools 3.0 provided by the HMMER server were utilized in this study [21] under default parameters to identify soybean WHY proteins. All potential WHY protein sequences underwent additional analysis using two online tools, namely the conserved domain database (CDD) available at https://www.ncbi.nlm.nih.gov/Structure/cdd/wrpsb.cgi (accessed on 20 December 2023) and the Simple Modular Architecture Research Tool (SMART) accessible via http://smart.embl-heidelberg.de (accessed on 20 December 2023). The soybean WHY proteins’ physical and chemical characteristics were determined by employing the ProtParam tool available on the ExPASY Bioinformatics Resource Portal (https://web.expasy.org/protparam/, accessed on 25 December 2023) [22]. The spatial distribution of *WHY* genes on the soybean chromosomes and the organization of *WHY* genes were visualized using TBtools (version 2.003) [23]. The subcellular localization of GmWHY proteins was predicted using the online tool CellPLoc 2.0, available at http://www.csbio.sjtu.edu.cn/bioinf/Cell-PLoc-2/ (accessed on 25 December 2023) [24].

### 2.2. Analysis of the Genomic Position, Arrangement, and Distribution of Conserved Domains in GmWHYs

The exon/intron location information of GmWHY genes was obtained from the annotation file of the soybean genome (version Gmax_Wm82_a2_v1). Protein domains and conserved motifs analysis were conducted using NCBI [25], MEME [26], and the websites as used in Krupinska et al. [1]. The TBtools software (version 2.003) [23] was utilized to generate graphical representations of chromosomal location, structure, conserved domains, and motifs. I-TASSER [27] was employed for predicting the protein structures of GmWHYs, which were then visualized with iCn3D (https://structure.ncbi.nlm.nih.gov/Structure/icn3d/ (accessed on 29 December 2023)).

### 2.3. Phylogenetic Investigations and Categorizations of the GmWHY Proteins

The muscle method of the MEGA_X_10.1.7 program with the default settings was used to perform multiple alignments of WHY protein sequences from *Arabidopsis* and soybean. A neighbor-joining (NJ) phylogenetic tree was constructed using MEGA_X_10.1.7 with 1000 bootstrap replicates and depicted by iTOL (https://itol.embl.de/ (accessed on 27 January 2024)). To classify the identified GmWHY proteins, we referred to records of AtWHY subfamily members in the TAIR database (https://www.arabidopsis.org/ (accessed on 27 January 2024)) and grouped them accordingly into different subfamilies.

### 2.4. Analysis of cis-Regulatory Elements in GmWHYs for Promoter Identification

The promoter regions of each *GmWHY* gene were obtained by extracting the genome sequence located 2 kb upstream. The cis-regulatory elements within these promoters were analyzed using PlantCARE (http://bioinformatics.psb.ugent.be/webtools/plantcare/html/, accessed on 30 December 2023) [28] and visualized using the TBtools software (version 2.003) [23].

### 2.5. Expression Analysis of GmWHYs in Tissues and under Low Phosphate Stress

The expression levels of the *GmWHY* genes in different tissues were obtained from the dataset published by Severin et al. This dataset provided comprehensive gene expression data for a wide range of fourteen tissues, including nodule, root, leaf, flower, pod, and pod shell at 10 and 14 DAFs (days after flowering), as well as seed at 10, 14, 21, 25, 28, 35, and 42 DAFs throughout the growth cycle of soybean [29]. The tissue expression heatmap was generated using the TBtools software (version 2.003) [23].

For the low Pi stress experiment, we followed the established procedure [30]. Williams 82 plants were cultivated in a controlled environment chamber with artificial climate conditions (28–33 °C/20–25 °C day/night temperature and 14 h light/10 h dark photoperiod). We used a half-strength Hoagland solution [2.5 mM Ca(NO_3_)_2_, 2.5 mM KNO_3_, 1.0 mM MgSO_4_, 0.5 mM KH_2_PO_4_, 10 μM EDTA Na_2_, 10 μM FeSO_4_, 23 μM H_3_BO_3_, 4.5 μM MnCl_2_, 0.15 μM CuSO_4_, 0.4 μM ZnSO_4_, and 0.05 μM Na_2_MoO_4_] as the normal Pi (NP) nutrient solution, and the low Pi (LP) nutrient solution was the same as the NP nutrient solution but with only 0.005 mM KH_2_PO_4_; the lack of KH_2_PO_4_ was replaced by an equal concentration of KCl. After hydroponic cultivation for a period of fifteen days under these conditions, leaf and root samples were collected from each plant replicate (three biological replicates in total). All samples were quick-frozen with liquid nitrogen and then stored at −80 °C for RNA isolation.

### 2.6. RNA Isolation and Reverse Transcription Quantitative PCR (RT-qPCR) Analysis

The SPARKeasy Plant RNA Extract Kit (SparkJade, Qingdao, China) was used to extract RNA according to the provided instructions. Subsequently, 1 µg RNA was reverse-transcribed using a SPARKscript II RT Plus Kit (With gDNA Eraser) (SparkJade, Qingdao, China). The expression levels of *GmWHY1*–*GmWHY7* were determined through RT-qPCR with *Tubulin* (GenBank accession number: AY907703) serving as a quantitative control. The specific primers can be found in Table S1. RT-qPCR analysis was conducted using 2 × SYBR Green qPCR Mix kit (SparkJade, Qingdao, China) on the CFX96 Touch system (Bio-Rad Laboratories, Hercules, CA, USA). The relative expression was calculated following the established methods [31].

### 2.7. Haplotype Analysis of WHYs

We conducted an in-depth investigation of the genomic region spanning the promoter and coding regions of seven *WHY* genes among 559 soybean accessions, which included 121 wild, 207 landrace, and 231 cultivated soybean accessions; their sequencing data and markers were obtained from Lu et al. [32]. We identified seven, five, fifteen, six, nine, five, and nineteen SNPs in *GmWHY1* to *GmWHY7*. The haplotypes of seven genes were analyzed with the Haploview4.1 software (https://sourceforge.net/projects/haploview/files/haploview-source/4.1/ (accessed on 29 February 2024)).

## 3. Results

### 3.1. Identification of WHY Gene Family in Soybean

To identify the *WHY* gene, we conducted a Hidden Markov Model (HMM) search on the soybean *Wm82.a2.v1* genome using the conserved domain WHY (PF08536). A total of seven *WHY* genes were identified in soybean. Based on their chromosomal locations and names, these seven *GmWHY* genes were named *GmWHY1*~*GmWHY7* (Table S2). The *GmWHY* family members were further characterized, encompassing the examination of the length of the coding DNA sequence (CDS), size of protein, molecular weight (MW) of protein, isoelectric point (pI), and anticipation of the subcellular localization. The lengths of the CDS for the *GmWHY* genes were between 381 bp and 810 bp, and *GmWHY2* and *GmWHY4* contained only one CDS, but *GmWHY1*, *GmWHY3*, *GmWHY5*, *GmWHY6*, and *GmWHY7* contained two, five, four, three, and two CDSs, respectively (Table S3). Most of the GmWHYs had a similar polypeptide, varying from 127 (GmWHY3) to 270 (GmWHY5) amino acid residues, corresponding to an MW between 14.61 kDa (GmWHY3) and 29.95 kDa (GmWHY5). The putative theoretical pI varied from 5.54 (GmWHY3) to 9.71 (GmWHY2). The online subcellular localization prediction showed that these GmWHYs were mainly distributed on the cell membrane, chloroplast, cytoplasm, nucleus, cell wall, and vacuole. All the data suggest that the *GmWHY* genes exhibit significant variability and fulfill distinct roles within various cellular compartments in soybean.

### 3.2. Phylogenetic Analyses of GmWHYs

**Figure 1:**
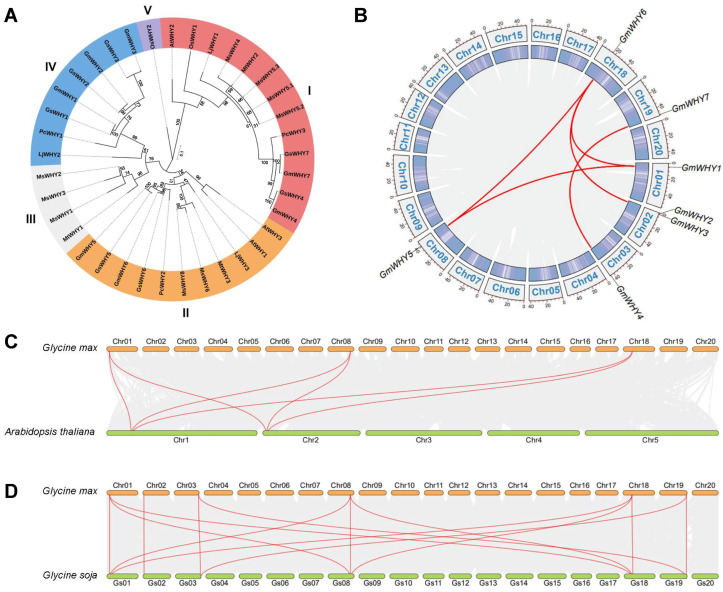
Comparison of the *WHY* gene family. (**A**) Phylogenetic analysis of the *WHY* gene family. The phylogenetic tree was constructed for the *WHY* gene family using 37 *WHY* genes from various plant species, including *A. thaliana* (At), *O. sativa* (Os), *M. truncatula* (Mt), *M. sativa* (Ms), *L. japonicus* (Lj), *P. coccineus* (Pc), *G. max* (Gm), and *G. soja* (Gs). The classification of the *WHY* gene family into subfamilies was denoted as I, II, III, IV, and V. (**B**) Positions and synteny of *GmWHY* genes. The duplicated *GmWHY* gene pairs were connected by red lines. (**C**) Synteny patterns of the *WHY* genes between *G. max* and *A. thaliana*. (**D**) Synteny patterns of the WHY genes between *G. max* and *G. soja*. The red lines were used to emphasize the syntenic *WHY* gene pairs between soybean and other species.

The *WHY* gene family contained seven members in soybean, seven members in *Glycine soja*, three members in *Phaseolus coccineus*, ten members in *Medicago sativa*, three members in *Medicago truncatula*, three members in *A. thaliana*, three members in *Lotus japonicas*, and two members in *Oryza sativa* (Table S4). In order to evaluate the evolutionary relationships among monocotyledonous and dicotyledonous plants, we selected the WHY amino acid sequences of the above eight plants for phylogenetic analysis. As shown in Figure 1A, these proteins were divided into five clades. Among these subfamilies, clade Ⅰ exhibited the highest membership count with a total of thirteen members, while the clade Ⅴ had the fewest, with one member. Clade Ⅳ was found exclusively in dicotyledonous plants among the seven species; clades Ⅰ, Ⅱ, and Ⅲ were shared by both monocotyledonous and dicotyledonous plants; clade Ⅴ was solely present in *O. sativa*. Among the seven GmWHYs, two, two, and three GmWHYs were clustered into clades Ⅰ, Ⅱ, and Ⅳ, respectively. This result suggests that *WHY* genes in monocotyledonous plants and dicotyledonous plants have both commonalities and characteristics, and the *WHY* gene families of monocotyledonous plants and dicotyledonous plants are ongoing evolutionary process.

### 3.3. Chromosome Localization and Collinear Relationship Analysis of GmWHY Genes

The seven *GmWHY* genes exhibited uneven distribution across all 20 chromosomes (Figure 1B). Chromosomes 1, 3, 8, 18, and 19 harbored *GmWHY1*, *GmWHY4*, *GmWHY5*, *GmWHY6*, and *GmWHY7*, respectively; chromosome 2 harbored *GmWHY2* and *GmWHY3*. The distribution among the gene subfamilies is relatively scattered, indicating a complex evolutionary history, and different genes may have undergone distinct evolutionary trajectories.

To explore the factors responsible for the increase in *GmWHY* gene members, an analysis of collinearity was conducted on the soybean genome. Our investigation into gene family duplication revealed the presence of five pairs of duplicated *GmWHY* genes within the soybean genome (Figure 1B). The *GmWHY* genes that were duplicated all belonged to segmental duplication events (Table S5). In addition, a comprehensive analysis was conducted on the *WHYs* in soybean and compared to those found in *A. thaliana* and *G. soja*. A total of 20 gene pairs that were orthologous were identified between soybean and the two other species (Figure 1C,D; Tables S6 and S7). The highest number of orthologous *WHY* gene pairs (including 14 pairs) was observed between soybean and *G. soja*, followed by 6 orthologous *WHY* gene pairs between soybean and *A. thaliana*.

Subsequently, we conducted estimations on the Ka, Ks, and Ka/Ks values of the *WHY* gene pairs (Tables S6 and S7), revealing that the most Ka/Ks ratios of the *WHY* homologous pairs were below one in *A. thaliana* and soybean, as well as in soybean and wild soybean (*G. soja*). These findings suggest that *GmWHYs* may have undergone significant purifying selective pressures during evolution, particularly between soybean and *Arabidopsis* as well as wild soybean and soybean, which likely played a crucial role in the survival and adaptation of soybean.

### 3.4. The Structure, Conserved Domain, and Motif Analyses of the GmWHYs

**Figure 2:**
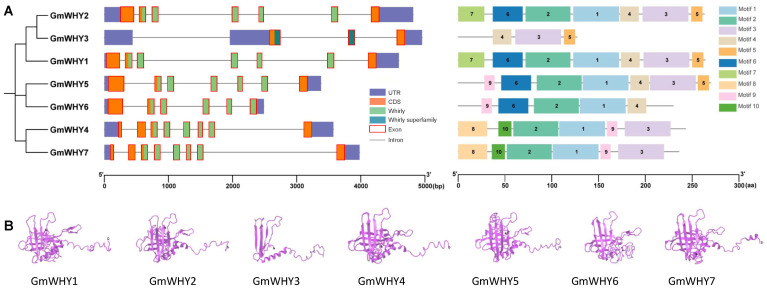
Structure of GmWHYs. (**A**) The phylogenetic tree, gene structure, domains, and motifs of GmWHYs. Left panel: exons and introns are represented by red color boxes and gray lines, respectively, and the domains are represented by differently colored boxes. Right panel: ten motifs are represented in differently colored boxes and were predicted by MEME [26]. The sizes of exons and introns are proportional to their sequence lengths. (**B**) Predicted protein structure of GmWHYs. N: and C represent the N-terminus and C-terminus of the protein, respectively.

To understand the structure of *GmWHYs*, members of *GmWHY* were acquired through an exploration of genomic DNA sequences from soybeans. *GmWHY* contained three or eight exons, with most of them having more than six exons (Figure 2A, Table S2). All GmWHY proteins contained the WHY domain, and ten conserved motifs (motifs 1–10) were analyzed in detail using the MEME online website (Figure 2A and Figure S1; Table S8). Additionally, all seven GmWHY protein contained the WHIRLY domain, GmWHY5/6 contained the DNA-binding motif (DBM, KGKAAL), GmWHY4/7 contained the cysteine domain (C), GmWHY2/5/6 contained the putative nuclear localization motif (pNLS), GmWHY4/7 contained the putative copper-binding motif (pCBM), and GmWHY1 to GmWHY5 and GmWHY7 contained the putative transactivation domain (pAD) (Table S9, Figure S1). Generally, the individual branches of the *GmWHY* gene exhibited similar structures in terms of genes, conserved domains, and motifs, but the different number of domains and motifs resulted in differently predicted protein structures (Figure 2B). These findings underscore its significance in biological processes across various organisms.

### 3.5. Analysis of cis-Regulatory Elements in GmWHYs

**Figure 3:**
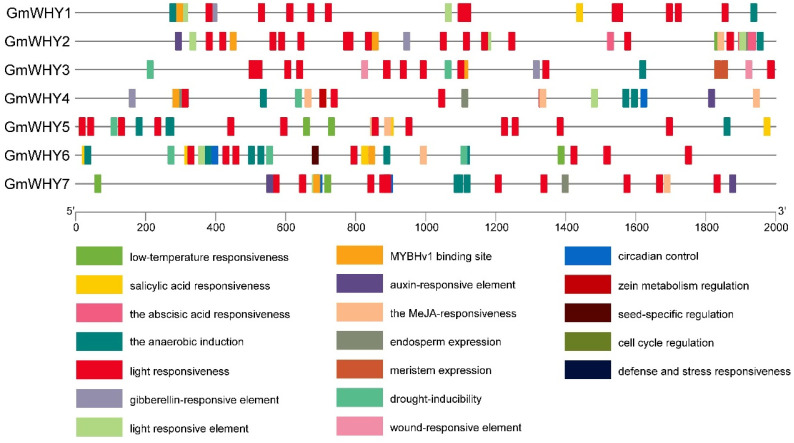
Cis-elements in the *GmWHY* gene promoter regions. The arrangement of the cis-regulatory elements within the 2000 bp upstream genetic regions of the seven identified *GmWHYs* is represented by colored boxes, each indicating a different cis-element.

To gain in-depth insights into the attributes of the *GmWHY* gene family, we performed an extensive investigation by acquiring and visually illustrating the 2000 bp sequences upstream of the start codon (ATG) for all *GmWHYs* in order to detect their *cis*-regulatory elements. Nineteen elements were identified in the promoter regions of the *GmWHY* genes (Figure 3, Table S10). These *cis*-regulatory elements mainly include four categories: elements associated with hormones (such as auxin, gibberellin, abscisic acid, methyl jasmonate, salicylic acid, etc.), elements responsive to light stimuli, elements related to stress responses (low temperature, wounds, oxygen deficit, etc.), and elements related to growth regulation (cell cycle regulation, endosperm expression, circadian control, etc.). For example, AUXIN RESPONSE FACTORs (ARFs) bind to the aux-response elements (AuxREs) in the promoter region of early auxin response genes and activate or repress their transcription, as reviewed by Guilfoyle and Hagen [33]; ARF7 and ARF19 regulate the lateral root formation via the direct activation of the LBD/ASL gene by binding the AuxRE of LBD/ASL in Arabidosis [34]; and LBD-mediated root development has been proved to be involved in the response to Pi deficiency in maize and white lupin [35,36]. These findings highlight the potential importance of *GmWHYs* in regulating various aspects of soybean growth and development.

### 3.6. The Genetic Diversity of GmWHY Genes among Soybean Accessions

**Figure 4:**
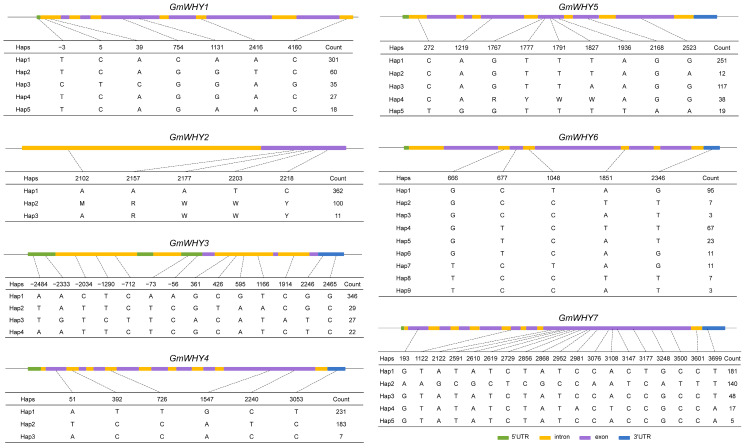
Haplotypes of *GmWHY* genes among the natural soybean population. Green, yellow, purple, and blue bars represent 5′UTR, introns, exons, and 3′UTR.

To understand the diversity of *GmWHYs* in nature soybean population, we analyzed the genetic variations of *GmWHYs* in 559 soybean accessions, which including 121 wild soybeans, 207 landraces, and 231 cultivated accessions [33]. The 7, 5, 14, 6, 9, 5, and 19 linked SNPs of *GmWHY1* to *GmWHY7* were divided into five, three, four, three, five, nine, and five haplotype groups, respectively (Figure 4). *GmWHY7* and *GmWHY3* contained the most genetic variations, but *GmWHY6* contained the most haplotypes. For *GmWHY2*, *GmWHY4*, *GmWHY5*, and *GmWHY7*, their SNPs were mostly located in exon regions, while for *GmWHY1*, *GmWHY3*, and *GmWHY6*, their SNPs were mostly located in intron regions.

### 3.7. Tissue Expression Pattern Analysis of GmWHY Genes

**Figure 5:**
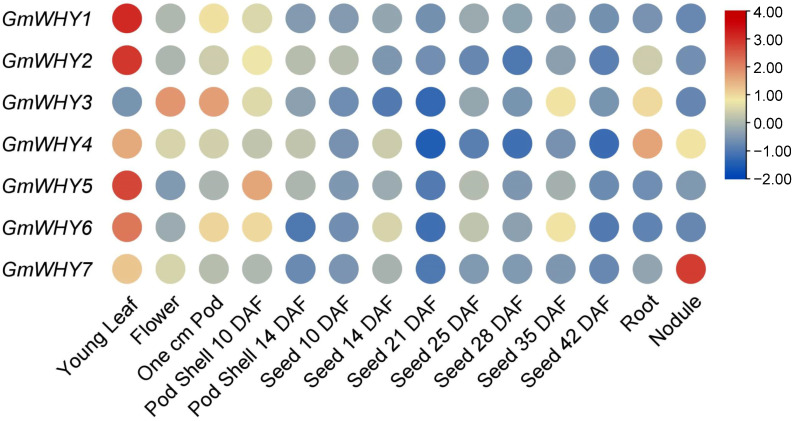
Analysis of *GmWHY* expression in various soybean tissues. Evaluation of *GmWHY* expression in different tissues during soybean development using publicly available RNA-seq data. The heatmap depicts log2-normalized RPKM values to represent gene expression levels. DAFs denote days after flowering.

To understand the expression of *GmWHYs* in the different tissues of soybean, we extracted the expression of *GmWHYs* from the transcriptome data in the soybean genome database and analyzed the expressions in different tissues. The expression levels of *GmWHY5* and *GmWHY6* were relatively high in various tissues, including leaves, pods, and seeds (Figure 5 and Table S11); other members exhibited high expression specifically in certain tissues, like leaves for *GmWHY1* and *GmWHY2*, flowers and pods for *GmWHY3*, leaves and roots for *GmWHY4*, and nodules for *GmWHY7*. Therefore, the diverse expression patterns observed in *GmWHYs* imply a non-specific role that is dependent on the tissue.

### 3.8. Expression Profiles of GmWHY Genes under Low Phosphate Stress

**Figure 6:**
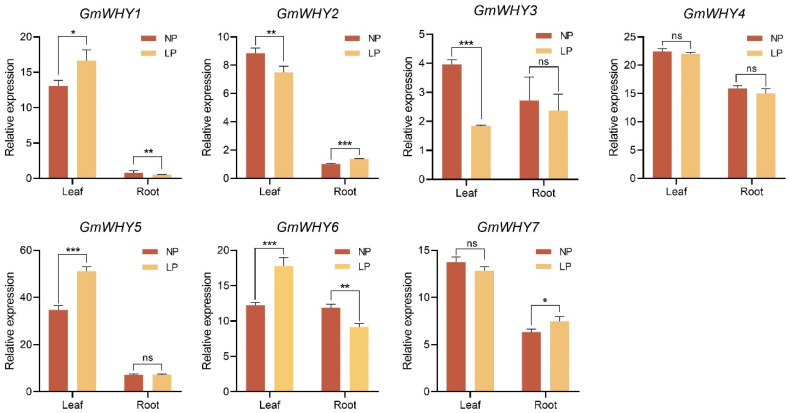
Expression analyses of *GmWHYs* in the leaves and roots of the cultivar Williams 82 under different Pi treatment levels. Data were normalized to the *GmTubulin* gene, and columns and error bars represent the means ± standard deviation (SD) of three independent biological replicates. Differences were evaluated using the two-tailed Student’s *t*-test (*** *p* < 0.001, ** *p* < 0.01, * *p* < 0.05, ns = no differences). LP, low Pi supply (5 μM, Pi); NP, normal Pi supply (500 μM, Pi).

Studies have shown that low Pi stress is one of the important factors limiting the growth and development of soybean [20,37]. In order to investigate whether *GmWHY* genes are involved in response to low Pi stress, the expression of *GmWHY* genes was detected in the leaves and roots of Williams 82 soybean accession using RT-qPCR under normal Pi (NP) and low Pi (LP) conditions. Five and four *GmWHYs* showed significant differences in the leaves and roots between NP and LP concentrations, respectively (Figure 6). In addition, *GmWHY1*, *GmWHY2*, and *GmWHY6* showed significant differences between NP and LP concentrations both in roots and leaves. The expression of some *GmWHYs* showed opposite trends after the LP treatment. These findings indicate that the involvement of *GmWHY* gene family members in addressing low Pi stress may be controlled by diverse molecular mechanisms.

## 4. Discussion

The WHY protein family is widely distributed in plants, and it serves various crucial functions in plant growth and resilience to environmental pressures [38]. WHY1 plays a crucial role in controlling the expression of genes responsible for encoding various essential proteins required for normal cellular functions and also regulates plant growth and development in response to both biological and environmental stresses [1]. It functions as a nuclear transcription factor, controlling the synthesis of hormones like ABA and SA [1,39]. Several studies have indicated that modifications in the abundance of WHYs in plants are linked to variations in the levels of reactive oxygen species (ROS) within organelles, thereby influencing their resistance towards abiotic stress. Under high light intensity, the RNAi-mediated knockdown of the *WHY1* gene can increase the levels of ROS in the chloroplasts of barley plants. The deficiency of *WHY1* in barley resulted in a delay in chloroplast development, leading to delayed greening and photosynthesis. This highlights the crucial role of *WHY1* in chloroplast biogenesis [15,40]. In addition, the older leaves of *why1* mutants also showed higher ROS levels in *A. thaliana* [41]. The overexpression of *WHY2* results in a reduction in ROS levels and an elevation of antioxidative enzyme activities in tobacco plants [42]. In this study, seven *WHY* genes were identified (Figure 1B) and had different subcellular locations in soybean (Table S2). These studies suggest significant differences in the WHY protein’s function. Understanding the underlying reasons for these variations is crucial in unraveling the complexities of biological systems.

Genes that exhibit distinct exon–intron arrangements and conserved domains may possess a wide range of functionalities [43]. In spite of their removal during post-transcriptional processing, introns have been hypothesized to possess significant significance in the evolution of plants and are regarded as a crucial mechanism for genes to acquire new functionalities [44]. Hence, it is imperative to perform a thorough examination of the exon–intron arrangement, conserved domains, and motifs within gene family members in order to investigate their evolutionary connections. In this study, *GmWHYs* were observed to contain three to eight exons; the number and arrangement of exons within a gene can have significant effects on its function and expression (Figure 2A). Understanding the structure and organization of *GmWHYs* is crucial for unraveling their biological roles in plant growth and development.

The outcomes of a phylogenetic tree analysis provide valuable insights into the relationship between organisms and their functional similarities. The clustering pattern observed in the phylogenetic tree reflects the evolutionary history of species, with closely related organisms being grouped together. This close clustering relationship often indicates a higher likelihood of having similar functions [45]. In this study, the phylogenetic tree constructed with WHY proteins specifically was divided into five subfamilies. By the subcellular localization analysis of soybean WHY proteins, GmWHY1, GmWHY2, and GmWHY5 were only located in the cell membrane, GmWHY4 was only located in the cell wall, and the other three GmWHY proteins were located in multiple sites of the cells (Table S2). The potential divergence in the subcellular distribution of WHY proteins suggest a possible variation in their respective functionalities. Additionally, segmental duplication events typically involve the dispersion of homologous genes across distant regions, whereas tandem duplication events occur when these genes are found in close proximity to each other [46]. According to our findings, the expansion of gene families is largely attributed to the process of gene duplication. Furthermore, the Ka/Ks ratios were found to be below 1 (Tables S6 and S7), indicating that the replication of the *GmWHY* genes is driven by selective purification and implying that the corresponding GmWHY proteins exhibit a relatively conserved characteristic.

Gene expression patterns play a crucial role in unraveling the intricate functions of genes [47]. By examining these patterns, scientists can gain valuable insights into how genes are regulated and their roles in various biological processes. In this study, the expression patterns of *GmWHYs* varied in different tissues. Most paralogous forms of this gene were found to be expressed in all tissues, while some exhibited tissue-specific expression or low expression (Figure 5). These differences may be attributed to the diverse functionalities or functional redundancy among *WHY* genes. As a vital nutrient element necessary for the growth and development of plants, Pi plays an indispensable role in acquiring, storing, and utilizing energy, regulating enzyme activities, and forming yields [20,48]. Therefore, we also investigated the expression of *WHY* family genes under low Pi stress. The expression of two *GmWHYs* in the leaves and three in the roots had no significant changes in soybean under low Pi stress, while other genes exhibited significant alterations. Among these significant alterative genes, the change tendency of different genes is also different, and the change in the same gene is different in different tissues (Figure 6). Under low Pi conditions, the expression levels of *GmWHY3* and *GmWHY5* in the leaves were significantly increased, while there was no significant change in the roots; the expression level of *GmWHY7* in the roots was significantly increased, while there was no significant change in the leaves under low Pi conditions. Hence, it is hypothesized that the involvement of the *GmWHY* gene family in managing low Pi stress could be plausible. Therefore, we hypothesized that certain genes belonging to the *WHY* family are implicated in soybean’s response to low Pi stress, and conducting a comprehensive investigation into their functionalities will play a pivotal role in elucidating the regulatory mechanism underlying plant Pi efficiency.

## 5. Conclusions

As plant-specific proteins, WHYs have a high impact on plant development and stress resistance. In this study, we discovered seven paralogous forms of a *GmWHY* gene that distributed on six chromosomes of soybean. We examined the gene structures, evolutionary relationships, promoter cis-acting elements, and expression patterns of *WHY* genes in soybean to investigate their phylogeny and diversification. The promoter region of *GmWHY* genes contains *cis*-regulatory elements associated with hormone regulation, stress response, targeted expression, and cell cycle regulation, among others. The collinearity analysis revealed that a substantial proportion of the *GmWHY* genes likely originated from segmental duplications, which may experience purifying selection due to their indispensable functions or advantageous roles in soybean evolution. The RT-qPCR analysis showed that six *GmWHY* genes responded differently to low Pi stress, which has important implications for improving soybean crop productivity. Understanding the role of *WHY* genes in agronomic traits, such as yield potential, nutrient uptake efficiency, and environmental stress tolerance, is important for the genetic improvement of Pi efficiency and yield of soybeans.

## Data Availability

Publicly available datasets were analyzed in this study. This data can be found here: (https://www.ncbi.nlm.nih.gov/geo/query/acc.cgi?acc=GSE173640, accessed on 25 December 2023)/GEO accession number: GSE173640.

## Supplementary Materials

The following supporting information can be downloaded at: https://www.mdpi.com/article/10.3390/genes15070833/s1. Figure S1: The motifs of seven GmWHY proteins. Motifs 1–10 were predicted by MEME [26]; DBM, WHIRLY, C, pNLS, pCBM, and pAD motifs were predicted using websites as reported in Krupinska et al. [27]. Table S1: Primers used in this study for RT-qPCR. Table S2: List of the identified *GmWHY* genes and their related information in soybean. Table S3: List of the coding sequences of *GmWHY* genes. Table S4: *WHY* genes in *Arabidopsis thaliana*, *Oryza sativa,* and legume plants. Table S5: Tandemly and segmentally duplicated *GmWHY* gene pairs. Table S6: One-to-one orthologous relationships between the WHY gene members in *Glycine max* and *Arabidopsis thaliana*. Table S7: One-to-one orthologous relationships between the *WHY* gene members in *Glycine max* and *Arabidopsis thaliana*. Table S8: Analyses of the motifs in *GmWHYs* from the MEME website. Table S9: The motifs in GmWHY proteins. Table S10: Cis-element analyses of the *GmWHY* gene promoter regions. Table S11: Expression profiles of *GmWHY* genes in multiple tissues throughout various developmental stages.
