# Supplementary figures and images for: Genome-Wide Identification of the Whirly Gene Family and Its Potential Function in Low Phosphate Stress in Soybean (Glycine max)
Source: Genes (Basel). 2024 Jun 25;15(7):833. doi: 10.3390/genes15070833 (PMC11275625; doi:10.3390/genes15070833)

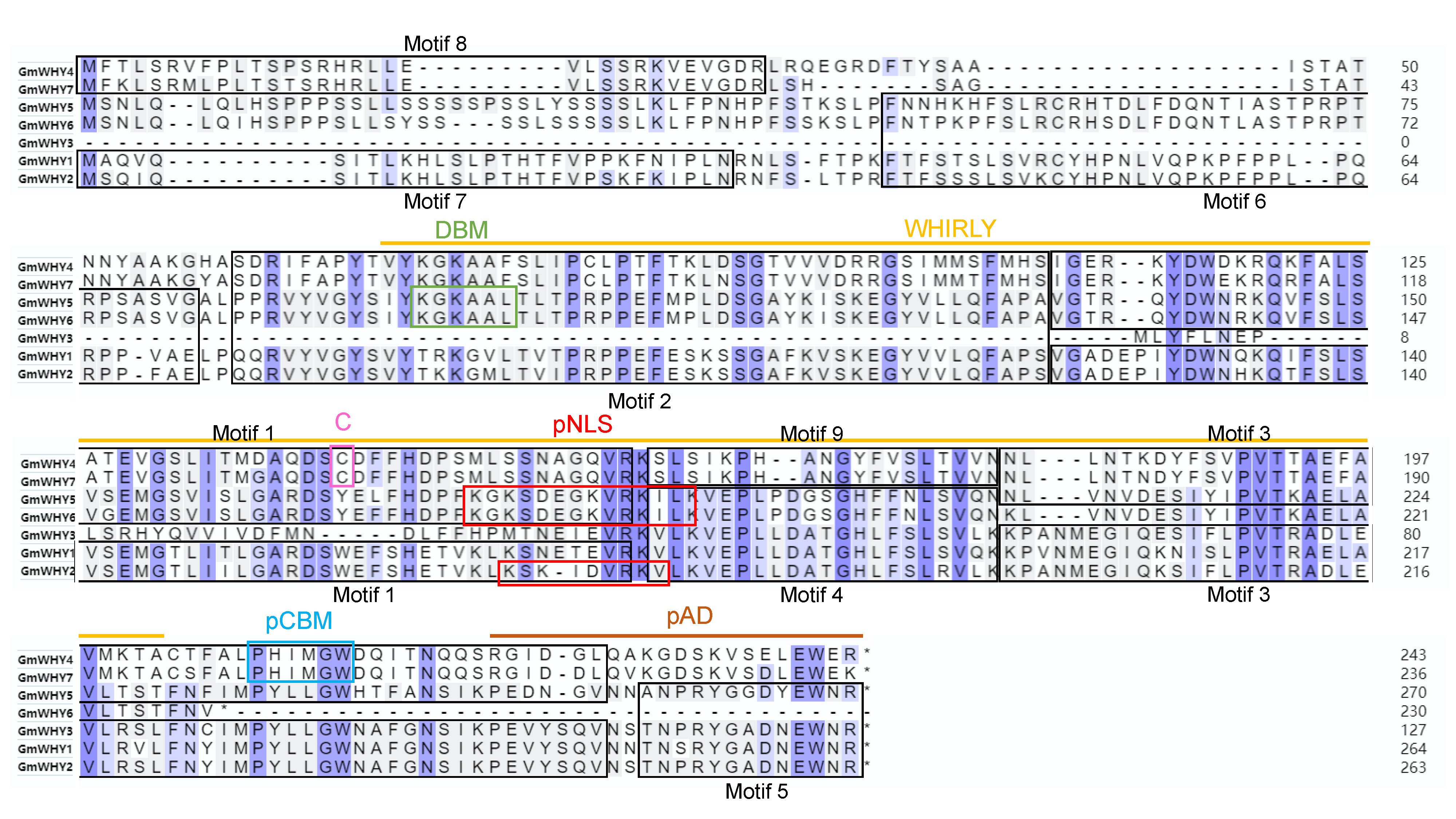

Supplement: Supplementary file 1 [file genes-15-00833-s001.zip › Figure S1.jpg]
